# Normalization of Rowland Universal Dementia Assessment Scale (RUDAS) in Chilean older people

**DOI:** 10.1590/1980-5764-DN-2023-0033

**Published:** 2023-12-11

**Authors:** Consuelo Sepúlveda-Ibarra, Fernando Henríquez Chaparro, Anthony Marcotti, Guillermo Soto, Andrea Slachevsky

**Affiliations:** 1 Universidad Bernardo O’Higgins, Facultad de Ciencias de la Salud, Escuela de Fonoaudiología, Santiago, Chile.; 2 Universidad de Chile, Facultad de Filosofía y Humanidades, Santiago, Chile.; 3 Geroscience Center for Brain Health and Metabolism, Santiago, Chile.; 4 Universidad de Chile, Faculty of Medicine, Neuropsychology and Clinical Neuroscience Laboratory, Physiopathology Program Institute of Biomedical Sciences, Neuroscience and East Neuroscience Departments, Santiago, Chile.; 5 Universidad de Chile, Faculty of Medicine, Hospital del Salvador Neurology Department, Memory and Neuropsychiatric Center, Santiago, Chile.; 6 Pontificia Universidad Católica de Chile, Facultad de Medicina, Laboratorio de Neurociencia Cognitiva y Evolutiva, Santiago, Chile.; 7 Universidad San Sebastián, Facultad de Odontología y Ciencias de la Rehabilitación, Escuela de Fonoaudiología, Santiago, Chile.; 8 Pontificia Universidad Católica de Chile, Facultad de Ciencias Sociales, Escuela de Psicología, Programa de Doctorado en Psicología, Santiago, Chile.; 9 Clínica Alemana-Universidad del Desarrollo, Departamento de Medicina, Servicio de Neurología, Santiago, Chile.

**Keywords:** Dementia, Aged, Literacy, Mental Status and Dementia Tests, Demência, Idoso, Alfabetização, Testes de Estado Mental e Demência

## Abstract

Rowland Universal Dementia Assessment Scale (RUDAS) is a cognitive screening that evaluates older people with low educational levels. In Chile, there are no normative data to assess this population.

**Objective:**

To obtain normative data on RUDAS in older Chilean people with up to 12 years of schooling, and to determine whether age and schooling years influence a person’s performance on RUDAS and on the items that constitute it.

**Methods:**

A group of cognitively healthy people 60 years old or over, with up to 12 schooling years was evaluated (n=135). Multiple regression models were applied to obtain normative data on RUDAS, according to age and schooling years, and to measure the effects of schooling on different items.

**Results:**

Regression analysis showed that none of the items had schooling as a significant predictor, except for the visuoconstruction item. The variables age and schooling explained 12.6% (R^2=0.126) of the RUDAS total score variance. The item visuoconstruction was the most associated with the educational level (OR=1,147).

**Conclusion:**

This study showed that RUDAS is a recommended instrument for evaluating older people with low educational levels. However, more studies are needed to prove the validity of the RUDAS on Chilean older people.

## RESUMO

RUDAS é uma triagem cognitiva que avalia idosos com baixa escolaridade. No Chile não existem dados normativos para avaliar essa população.

**Objetivo:**

Obter dados normativos sobre RUDAS em idosos chilenos com até 12 anos de escolaridade. Além disso, determinar se a idade e a escolaridade influenciam o desempenho de uma pessoa no RUDAS e nos itens que o constituem.

**Métodos:**

Foi avaliado um grupo de pessoas cognitivamente saudáveis, com 60 anos ou mais e até 12 anos de escolaridade (n=135). Modelos de regressão múltipla foram utilizados para obter dados normativos do RUDAS, segundo idade e anos de escolaridade, e para mensurar os efeitos da escolaridade em diferentes itens.

**Resultados:**

A análise de regressão mostrou que nenhum dos itens teve a escolaridade como preditor significativo, exceto o item visuoconstrução. As variáveis idade e escolaridade explicam 12,6% (R^2=0,126) da variância do escore total do RUDAS. O item visuoconstrução é o mais associado ao nível de escolaridade (OR=1,147).

**Conclusão:**

Este estudo mostrou que o RUDAS é um instrumento recomendado para a avaliação de idosos com baixa escolaridade. No entanto, são necessários mais estudos para comprovar a validade do RUDAS em idosos chilenos.

## INTRODUCTION

Dementia is a public health problem. It is estimated that by 2050 it will affect 152 million people, with a higher proportion in low-income countries^1^. Multiple risk factors increase the probability of developing dementia, including a low educational level^2^, which is not only a significant risk factor but also has a higher rate of sub-diagnoses^3^, and is even higher in low-income countries^4^. Currently, there are few screening instruments to evaluate this population^5^.

Dementia diagnosis is complex, and timely detection is essential to initiate medical care and delay its impact on people’s functionality. Furthermore, it is important to minimize the effects on caregivers and improve patients’ quality of life^6^. The diagnosis is established based on clinical symptoms and cognitive evaluation, with the objectification of cognitive decline serving as a criterion to determine the condition^7^. It is also known that educational level is one of the main factors affecting cognitive test performance, justifying the need to improve evaluation methods for illiterate people and those with low educational level^8^.

The Rowland Universal Dementia Assessment Scale (RUDAS) was designed to assess people with low educational levels^9^. It was developed in Australia and currently has validation in different countries^10-20^, where it obtained good psychometric measures that suggest its usefulness. Its results observed a low influence of schooling on its total score in several studies^21^. Nonetheless, this continues to be a subject of research. It has been recommended for public health use due to its easy and quick application^22^.

In Latin America (LA), there are validations in Peru and Brazil, and so far, RUDAS has not been normalized in Chile. In this country, 8.2% of the elderly population over 60 years old is illiterate^23^, which requires adequate instruments for evaluation.

The normalization of a test refers to a collection of scores from a representative sample of the general population and, in general, a relatively large sample size is used^24^. To avoid the need for a large sample size, this paper proposes a normalization based on a regression model, which allows the obtention of valid normative data with small sample of subjects^25^.

This investigation aims to obtain normative data from RUDAS on older Chilean people with up to 12 years of schooling, in addition to determining if age and years of education influence a person’s performance in this cognitive screening and the items that constitute it.

## METHODS

An observational cross-sectional study was designed. The study protocol was approved by the Scientific Ethics Committee of Facultad de Medicina of Universidad de Chile, under 168-2018. All subjects signed their corresponding informed consent.

### Participants

A non-probabilistic sampling was carried out, extending an open invitation to participants of elderly clubs and beneficiaries of Programa Vínculos, financed by Servicio Nacional del Adulto Mayor (SENAMA). This national public policy aims to increase the active participation of older people in their communities, enabling access to social conditions improvements^26^.

The participants had to meet the following inclusion criteria: be 60 years old or over, have between 0 and 12 schooling years, have functional hearing and vision, or, if necessary, have technical aids in good condition (hearing aids, optical lenses), and not having difficulties in their daily living activities measured by the technology-activities of daily living questionnaire (T-ADLQ)^27^.

The study excludes participants with cognitive alterations, with less than 13 points in the abbreviated Mini Mental State Examination (MMSE)^28^. It also excludes participants with depressive symptoms, with a score >2 in the Patient Health Questionnaire version 9 (PHQ-9)^29^ and a score >2 in the dementia screening questionnaire - Chilean version (AD8-Ch)^30^, participants with a history of drug use and alcohol abuse, with a total score in the Alcohol Use Disorder Identification Test (AUDIT-C)^31^ >4, and a diagnosis of uncontrolled chronic non-communicable diseases with medicine.

**Figure 1.**
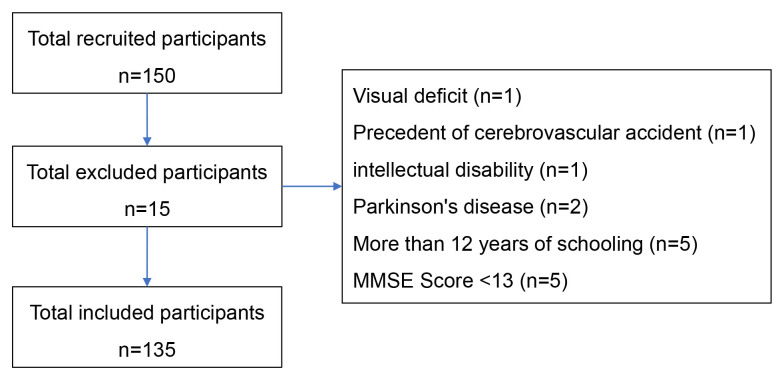
Flowchart of the study participants.

One hundred and fifty subjects answered the invitation. Within those 150 respondents, 15 were excluded: one for presenting visual deficit without technical assistance, one for having a precedent of cerebrovascular accident, one for being diagnosed with intellectual disability, two for being diagnosed with Parkinson’s disease, five for having more than 12 years of schooling, and another five for having a low performance in the abbreviated MMSE. The final sample consisted of 135 participants (Figure 1).

### Instruments and procedures

Each participant and their companion were evaluated in a single session. The study’s objective was explained, and they were requested to sign informed consent. A brief interview was conducted, and the tests mentioned above were administered to corroborate compliance with inclusion and exclusion criteria. After confirming their suitability for the study, a RUDAS Spanish version was applied (Supplementary Material: https://www.demneuropsy.com.br/wp-content/uploads/2023/09/DN-2023.0033-Supplementary-Material-1-e-2.docx). The session was conducted by an evaluator in a distractor-free room, and it lasted approximately 30 minutes.

### Instrument description

RUDAS is a cognitive screening test that is easy to apply and takes little time (12.8 minutes in its Spanish version). It evaluates six cognitive domains: memory, body orientation, praxis, visuoconstruction, judgment, and language. It grants a total score ranging from 0 to 30, with 23 being the cut-off score in the original version^9^ and 21/22 in its Spanish version^10^.

In its original version, RUDAS presented a receiver operating characteristic (ROC) curve of 0.94, 95% confidence interval (CI), 89% sensitivity (95%CI 76–96%), and 98% specificity (95%CI 88–97%).

### Statistical analysis

The analysis was conducted using Stata Statistics v16.1 software. For descriptive purposes, means and standard deviations (SD) were estimated for the quantitative variables, and absolute and relative frequencies were estimated for the qualitative variables. The Shapiro-Wilk test was applied to investigate the data distribution. To select the covariates, relationships with the RUDAS total score were explored using a *t*-test for independent samples or Spearman’s correlation coefficient (rho), depending on the nature of each one.

To estimate the normative values, a regression-based normalization^32^ was performed using the conditional mean to determine the expected score for each possible combination of covariates. The assumptions of a linear relationship, homoscedasticity, multicollinearity, and normality of the residuals were checked. Finally, ordered logistic regression models were created to perform an analysis of the schooling influence on RUDAS for each item. The corresponding odds ratios (OR) were estimated, and a likelihood ratio test was used to determine the assumption of proportional odds.

## RESULTS

Of the 135 participants, 103 (76.3%) were women, while 32 (23.7%) were men. No differences were observed in age (t=0.580; p=0.562), schooling (t=0.480; p=0.632) or location (χ^2^(1)=1.124; p=0.289) between men and women. Regarding location, 100 (74.1%) belonged to the rural district of Calera de Tango, and 35 (25.9%) to the urban districts of Santiago, Pedro Aguirre Cerda, and Conchalí. No differences were observed in schooling according to location (t=-1.386; p=0.168). However, there were age differences (t=-2.906; p=0.004), with an average of 70.5 (SD 7.6) years for subjects from rural locations and 74.7 (SD 6.4) for subjects from urban locations. Table 1 illustrates the details of the descriptive statistics of the sample. Table 2 shows the descriptive statistics for each item of the RUDAS and its total score.

**Table 1.** Descriptive statistics of the sample.

Variable		n=135
Age (mean; ±SD)		71.6 (±7.5)
Sex (n; %)	Female	103 (76.3)
	Male	32 (23.7)
Years of education (mean; ±SD)		7.17 (±7.1)
Illiteracy (n)		2
Low – under 6 years (n)		61
High – above 6 years (n)		72
Locality (n; %)	Urban	35 (25.9)
	Rural	100 (74)
Chronic pathologies (n; %)	Hypertension	92 (68.1)
	Diabetes	30 (22.2)
	Hypothyroidism	12 (8.9)
	History of depression	27 (20.0)

Abbreviations: SD, standard deviation.

**Table 2.** Descriptive statistics of the RUDAS total score and its items (total sample: n=135).

Variable (RUDAS item)	Mean	SD	Median	IQR
Body orientation			5.00	0.00
Praxis			2.00	0.00
Visuospatial construction			1.00	3.00
Judgment	2.11	1.19		
Memory			6.00	2.00
Language			8.00	0.00
Total scoring	24.50	2.59		

Abbreviations: RUDAS, Rowland Universal Dementia Assessment Scale; SD, standard deviation; IQR, interquartile range. Notes: Mean and standard deviation were reported for normally distributed variables (p>0.05), while median and interquartile range were reported for variables with skewed distribution, as determined by the Shapiro-Wilk test.

### Selection of predictor variables

To select the regression model predictor variables, the relationship of the RUDAS total score with each of the variables of interest was studied independently. Age had a significant correlation with RUDAS total score (rho=-0.268; p=0.001), as well as schooling (rho=0.241; p=0.004). There were no significant differences between men and women (t=-0.068; p=0.945), and there were no differences between urban and rural locations (t=-0.103; p=0.917). Due to significant age differences evidenced by location, a preliminary multiple linear regression model was adjusted to assess the effect of location on the RUDAS total score controlled by age, resulting in a coefficient of 0.468 (95%CI -0.533–1.470) that was not a significant predictor (p=0.357).

### Estimation of normative values

A multiple linear regression was run, considering the RUDAS total score as the dependent variable and age and schooling variables as predictors. A significant regression equation was evidenced with F(2,132)=9.53, a p=0.000, and R^2=0.126. Both predictor variables were significant (Table 3). A significant constant (p<0.001) was also obtained with a value of 29.558 (95%CI 25.430–33.685). In this way, the expected score for a subject is estimated according to the following equation: Ŷ=β_0_+ β_1_*X_1_– β_1_*X_2_; where Ŷ is the expected score; β0 is the constant of the model; β1, the estimated coefficient for schooling; and β2, the estimated coefficient for age. The terms X1 and X2 correspond to schooling and age, respectively, both considered in years. In addition, it is possible to determine the degree of variation between the observed and expected values in terms of standard deviation by computing the difference between both and dividing it by the SD of the residuals. This standard error was 2.407 (95%CI 2.405–2.410). The normative data with their respective SD can be found in Supplementary Material 2 (https://www.demneuropsy.com.br/wp-content/uploads/2023/09/DN-2023.0033-Supplementary-Material-1-e-2.docx).

**Table 3.** Result of the multivariate linear regression model for the RUDAS total score.

	Coefficient	SE	95%CI		p-value
			LL	UL	
Age	-0.089	0.027	-0.144	-0.034	0.002
Education	0.190	0.067	0.057	0.323	0.005

Abbreviations: SE, standard error; CI, confidence interval; LL, lower limit; UL, upper limit.

### Analysis by item

**Figure 2.**
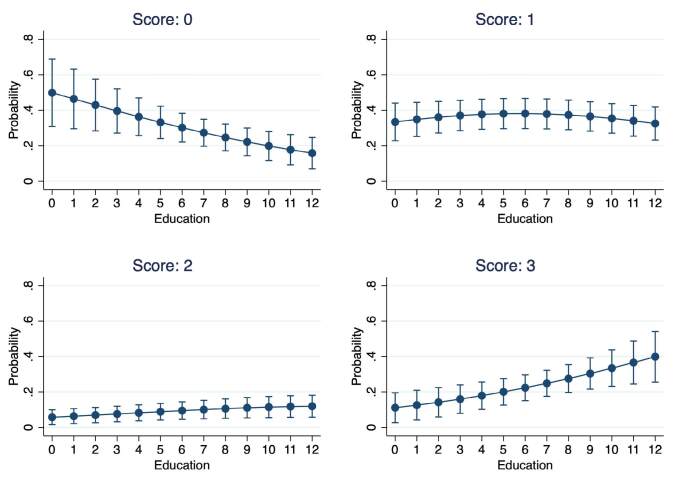
Association of years of education with the visuoconstruction item score.

Finally, ordered logistic regression models were created with the score of each RUDAS item as the response variable, and schooling was considered as the predictor variable. All models were adjusted to the age of the subjects. None of the items had schooling as a significant predictor, except for the visuoconstruction item (Table 4). For this item, the model obtained was significant with p=0.001, an LR chi^2(2)=13.48, a pseudo R^2=.038, and schooling obtained OR=1.147. That is to say, for each additional year of schooling, the probability of moving from one scoring category to another increases by 14.7%. The likelihood ratio test obtained was chi^2(4)=7.550 and p=0.109, meaning the estimation is the same for all scoring categories. Figure 2 shows the relationship between schooling and each of the possible scores for the visuoconstruction item.

**Table 4.** Result of the ordered logistic regression model for the score of the RUDAS items.

RUDAS items	OR	SE	95%CI		p-value
			LL	UL	
Body orientation	1.020	0.081	0.871	1.194	0.801
Praxis	1.054	0.071	0.923	1.205	0.432
Visuospatial construction	1.147	0.058	1.037	1.268	0.007
Judgment	1.054	0.054	0.952	1.167	0.303
Memory	1.025	0.055	0.923	1.139	0.635
Language	1.273	0.262	0.850	1.908	0.241

Abbreviations: RUDAS, Rowland Universal Dementia Assessment Scale; OR, odds ratio; SE, standard error; CI, confidence interval; LL, lower limit; UL, upper limit.

## DISCUSSION

In this article, the effects of age and schooling on RUDAS performance were determined along with RUDAS normative values calculation in cognitively healthy people 60 years of age or older, between 0 and 12 years of schooling, and living in different districts of the metropolitan region in Santiago, Chile. Results showed that age and schooling variables explain only 12.6% (R^2=0.126) of RUDAS total score variance. It was observed that the visuoconstruction item is the one most associated with the schooling variable (OR=1.147).

Validation papers carried out to this date in LA^14,18,19^ revealed the usefulness of RUDAS in the region. However, to our understanding, there is no normalization study in LA. The methodology of our normalization study provides a reference standard that can compare the performance of those evaluated and determine whether it is within normal limits^33^.

Although the influence of education level on RUDAS total score has been previously analyzed, there is only one normalization study conducted in Western Europe^34^. Similar to what was found in this investigation, the authors reported that the schooling variable explained 16% of RUDAS score variance (R^2=0.160), while other variables such as age and sex accounted for 3% (R^2=0.030) and 1% (R^2=0.010), respectively. On the other hand, in validation studies, the influence of educational level continues to be investigated. No influence of schooling years was reported in the original paper of Storey et al.^9^ and in LA^14,18.19^. However, in the review by Komalasari et al.^35^, a positive schooling effect was reported in five validation papers.

**Figure 3.**
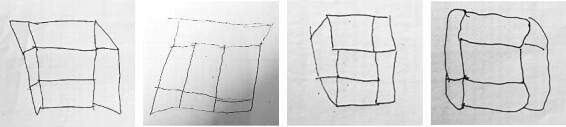
Examples of errors in the visuospatial construction item.

Regarding the visuoconstruction item, results suggest that this is one of the most associated with educational level. An influence was observed related to schooling on the cube copy test (OR=1.147). In the Arabic validation of RUDAS^13^, it was found that 51% of the participants without formal education could not copy the cube, while as the years of schooling increased, there was a higher rate of success in the task. In our investigation, 27.4% (n=37) obtained 0 points in the task, of which 75.6% (n=28) had eight or fewer years of schooling, and 70.2% (n=26) lived in urban districts. Similarly, the study carried out in Peru^36^ showed that illiterate people residing in rural districts performed significantly worse on this test than their peers living in urban districts. Figure 3 shows some examples of the errors that occurred in the task. The explanation for this difficulty is that people with no knowledge of geometry visualize the cube as a superposition of lines in a two-dimensional way^37^. Furthermore, the copy of the cube task depends not only on visuoconstructive skills but also on other cognitive functions such as semantic memory, attention, and organization, among others^38^. These functions are also favored by schooling years.

Different investigations analyze the influence of educational level on the cube copy test^39,40^. A recent study^41^ compared the diagnostic accuracy of the clock drawing, the copy of the cube, and the one with intertwined infinity signs. It determined that only the first test had a discriminating capacity between the control group vs. dementia, mild cognitive impairment (MCI) vs. dementia, and control group vs. cognitive impairment (MCI and dementia). The cube copy could only discriminate between controls and people with dementia. Based on the evidence presented in further studies, it would be important to investigate the diagnostic utility of this item in the evaluation of older people with low educational levels included in RUDAS and the relevance of proposing another visuoconstruction task as an alternative that provides greater precision to the instrument.

Unlike the previous item, the other items did not show an influence of education. However, it is important to highlight that the body orientation, praxis, and language items exhibited a pronounced ceiling effect, with at least 75% of the sample achieving the maximum score and showing minimal variability. This explains why the interquartile range (IQR) for these items was 0 and the median was the maximum score. These results can be attributed to the low difficulty of the items, and the high concentration of scores is consistent with the lack of variability contributed by age and education.

MMSE continues to be the most widely used cognitive screening in clinical practice. However, bias associated with education level and sociocultural factors has been observed^42^. RUDAS includes the evaluation of frontal functions (verbal fluency, judgment, and visuoconstruction), which is an advantage compared to MMSE because this could help to detect other dementias different from Alzheimer’s disease and also early changes in people with MCI^19,43^. On the other hand, the answer’s modality in all MMSE items is verbal, while in RUDAS, there are verbal, non-verbal, and written responses, which allows for a more complete evaluation of the person’s general cognitive functioning. In the systematic review with meta-analysis performed by Nielsen & Jørgensen^21^, it was observed that, in low- and middle-income countries, RUDAS results are less affected by schooling levels than MMSE. This observation regarding MMSE has also been found in its comparison with other instruments^44^.

The use of RUDAS has been recommended in primary health care (PHC)^22^ due to its characteristics of being an easy and quick administration instrument. The average application time in this study was 5 minutes and 76 seconds, which is lower than the average reported in other works^10,45,46^. In the validation made in Spain^10^, medical students were trained to conduct the evaluation, which required only brief instruction before sample collection. Similarly, in the original version, 40 minutes were taken to train health professionals who would perform the evaluation based on videos. This is an important benefit considering its possible use in PHC.

Concerning the methodology used to obtain the normative values, it supposes the overcoming of two great difficulties compared to classic normalization methods. First, these methods require using one or more significant covariates (for example, sex, age, or education) to define different subgroups and, finally, estimate the conditional distribution of raw scores and their statisticians for each one. However, this implies that continuous variables are used as categorical variables to establish groups^47^. A problem arising from this is the cohort edge effect^48^, in which two subjects with minimal age differences but with the same score can be assigned to different subgroups. Regression-based normalization allows the inclusion of covariates without having to be categorized, avoiding this effect^32^.

Second, classical methods require large sample sizes to make accurate estimates. Each of the defined subgroups requires a homogeneous number of observations; the more subgroups are established based on the covariates, the larger the sample size requirement. Given the nature of regression analyses, values are estimated using the complete sample. In addition, it considers all the possible values that the selected covariates could take, which translates into a smaller number of subjects needed to make estimates^25^.

The limitations of this work are that there is little data related to illiterate people, a low proportion of men and of people living in urban areas. Nevertheless, the sample was obtained in a community environment which ensures a certain representativeness of the general population. In light of the national demographic data, evaluating older people residing in the southern regions of Chile — La Araucanía, Ñuble, and/or Los Lagos — where the largest rural population is concentrated would constitute a contribution since rurality has been associated with an increased risk of MCI and a determinant of dementia^49^. Another contribution would be conducting evaluations in the regions of Valparaíso and Bío-Bío, two regions with the highest population density and the largest number of people over 64 years old, after the capital^50^.

This research’s findings suggest that RUDAS is an alternative to use in older people with 0 to 12 years of schooling. Therefore, future work should focus on the validation of the instrument in clinical population. This is to ensure the psychometric measures of validity in the Chilean population and thus to be able to establish a comparison with what was obtained in other LA countries. It would be relevant to know its diagnostic usefulness in people with schooling over 12 years and to compare its diagnostic accuracy with other frequently used instruments, such as the MMSE, for the investigation of MCI and dementia in PHC and other levels of public health care. This information would be useful to strengthen the evaluation processes currently carried out in the different programs and public policies linked to the early detection of MCI and dementia in the country.

## References

[1] GBD 2019 Dementia Forecasting Collaborators. (2022). Estimation of the global prevalence of dementia in 2019 and forecasted prevalence in 2050: an analysis for the Global Burden of Disease Study 2019. Lancet Public Health..

[2] Livingston G, Huntley J, Sommerlad A, Ames D, Ballard C, Banerjee S (2020). Dementia prevention, intervention, and care: 2020 report of the Lancet Commission. Lancet..

[3] Lang L, Clifford A, Wei L, Zhang D, Leung D, Augustine G (2017). Prevalence and determinants of undetected dementia in the community: a systematic literature review and a meta-analysis. BMJ Open..

[4] Nakamura AE, Opaleye D, Tani G, Ferri CP. (2015). Dementia underdiagnosis in Brazil. Lancet..

[5] Calil V, Elliott E, Borelli WV, Barbosa BJAP, Bram J, Silva FO (2020). Challenges in the diagnosis of dementia: insights from the United Kingdom-Brazil Dementia Workshop. Dement Neuropsychol..

[6] Aranda MP, Kremer IN, Hinton L, Zissimopoulos J, Whitmer RA, Hummel CH (2021). Impact of dementia: health disparities, population trends, care interventions, and economic costs. J Am Geriatr Soc..

[7] American Psychiatric Association. (2014). Manual diagnóstico y estadístico de los trastornos mentales DSM-5..

[8] Magklara E, Stephan BCM, Robinson L. (2019). Current approaches to dementia screening and case finding in low- and middle-income countries: research update and recommendations. Int J Geriatr Psychiatry..

[9] Storey JE, Rowland JTJ, Basic D, Conforti DA, Dickson HG. (2004). The Rowland Universal Dementia Assessment Scale (RUDAS): a multicultural cognitive assessment scale. Int Psychogeriatr..

[10] Ramos-Ríos R, Mateos-Álvarez R, López-Moríñigo JD. (2009). Cribado de demencia en una población con un bajo nivel de instrucción. Validación de la versión española del RUDAS (Rowland Universal Dementia Assessment Scale) en una muestra asistencial. Psiogeriatria..

[11] Nielsen TR, Andersen BB, Gottrup H, Lützhøft JH, Høgh P, Waldemar G. (2013). Validation of the Rowland Universal Dementia Assessment Scale for Multicultural Screening in Danish Memory Clinics. Dement Geriatr Cogn Disord..

[12] Juwita S, Aniza AA, Zorina A, Asrene AR. (2013). Validation of the Malay Version of Rowland Universal Dementia Assessment Scale (M_RUDAS) among elderly attending primary care clinic. International Medical Journal..

[13] Chaaya M, Phung TKT, El Asmar K, Atweh S, Ghusn H, Khoury RM (2016). Validation of the Arabic Rowland Universal Dementia Assessment Scale (A-RUDAS) in elderly with mild and moderate dementia. Aging Ment Health..

[14] Araujo NB, Nielsen TR, Engedal K, Barca ML, Coutinho ES, Laks J. (2018). Diagnosing dementia in lower educated older persons: validation of a Brazilian Portuguese version of the Rowland Universal Dementia Assessment Scale (RUDAS). Braz J Psychiatry..

[15] Ayan G, Afacan C, Poyraz BC, Bilgic O, Avci S, Yavuzer H (2019). Reliability and validity of rowland universal dementia assessment scale in Turkish population. Am J Alzheimers Dis Other Demen..

[16] Nepal GM, Shrestha A, Acharya R. (2019). Translation and cross-cultural adaptation of the Nepali version of the Rowland universal dementia assessment scale (RUDAS). J Patient Rep Outcomes..

[17] Chen CW, Chu H, Tsai CF, Yang HL, Tsai JC, Chung MH (2015). The reliability, validity, sensitivity, specificity and predictive values of the Chinese version of the Rowland Universal Dementia Assessment Scale. J Clin Nurs..

[18] Custodio N, Montesinos R, Lira D, Herrera-Perez E, Chavez K, Hernandez-Córdova G (2019). Validation of the RUDAS in patients with a middle-level education in Lima, Peru. Am J Alzheimers Dis Other Demen..

[19] Custodio N, Montesinos R, Lira D, Herrera-Perez E, Chavez K, Reynoso-Guzman W (2020). Validation of the RUDAS for the identification of dementia in illiterate and low-educated older adults in Lima, Peru. Front Neurol..

[20] Al-Jadidi S, Alharrasi M, Almaqbali M, Alkharusi F, Al Kharusi N, Al-Tai N (2022). The usefulness of rowland universal dementia assessment scale in Oman: a cross sectional study. Int J Geriatr Gerontol..

[21] Nielsen TR, Jørgensen K. (2020). Cross-cultural dementia screening using the Rowland Universal Dementia Assessment Scale: a systematic review and meta-analysis. Int Psychogeriatr..

[22] Coelho-Guimarães N, Garcia-Casal JA, Díaz-Mosquera S, Álvarez-Ariza M, Martínez-Abad F, Mateos-Álvarez R. (2021). Validación del RUDAS como instrumento de cribado de población con demencia en atención primaria. Aten Primaria..

[23] Chile. Ministerio de Desarrollo Social y Familia. (2017). Encuesta de caracterización socioeconómica nacional [Internet]..

[24] Zimmerman ME., Kreutzer JS, DeLuca J, Caplan B (2011). Encyclopedia of clinical neuropsychology..

[25] Oosterhuis HEM, van der Ark LA, Sijtsma K. (2016). Sample size requirements for traditional and regression-based norms. Assessment..

[26] Servicio Nacional del Adulto Mayor. Programa vínculos [Internet]..

[27] Muñoz-Neira C, López OL, Riveros R, Núñez-Huasaf J, Flores P, Slachevsky A. (2012). The technology – activities of daily living questionnaire: a version with a technology-related subscale. Dement Geriatr Cogn Disord..

[28] Chile. Programa de Salud del Adulto Mayor. División de Prevención y Control de Enfermedades. Subsecretaria de Salud Pública. Manual de aplicación del examen de medicina preventiva del adulto mayor [Internet]..

[29] Diez-Quevedo C, Rangil T, Sanchez-Planell L, Kroenke K, Spitzer RL. (2001). Validation and utility of the patient health questionnaire in diagnosing mental disorders in 1003 general hospital Spanish inpatients. Psychosom Med..

[30] Muñoz C, Núñez J, Flores P, Behrens MIP, Slachevsky A. (2010). Utilidad de un cuestionario breve dirigido al informante para el diagnóstico temprano de casos de demencia: la versión Chilena del AD8 (AD8-Ch). Rev Méd Chile..

[31] Alvarado ME, Garmendia ML, Acuña G, Santis R, Arteaga O. (2009). Validez y confiabilidad de la versión chilena del Alcohol Use Disorders Identification Test (AUDIT). Rev Méd Chile..

[32] Lenhard A, Lenhard W, Suggate S, Segerer R. (2018). A continuous solution to the norming problem. Assessment..

[33] Berrigan LI, Fisk JD, Walker LAS, Wojtowicz M, Rees LM, Freedman MS (2014). Reliability of regression-based normative data for the oral symbol digit modalities test: an evaluation of demographic influences, construct validity, and impairment classification rates in multiple sclerosis samples. Clin Neuropsychol..

[34] Nielsen TR, Segers K, Vanderaspoilden V, Bekkhus-Wetterberg P, Bjørkløf GH, Beinhoff U (2019). Validation of the Rowland Universal Dementia Assessment Scale (RUDAS) in a multicultural sample across five Western European countries: diagnostic accuracy and normative data. Int Psychogeriatr..

[35] Komalasari R, Chang HCR, Traynor V. (2019). A review of the Rowland Universal Dementia Assessment Scale. Dementia (London)..

[36] Custodio N, Montesinos R, Diaz MM, Herrera-Perez E, Chavez K, Alva-Diaz C (2021). Performance of the Rowland Universal Dementia Assessment Scale for the detection of mild cognitive impairment and dementia in a diverse cohort of illiterate persons from rural communities in Peru. Front Neurol..

[37] Teng EL. (2002). Cultural and educational factors in the diagnosis of dementia. Alzheimer Dis Assoc Disord..

[38] Shulman KI. (2000). Clock-drawing: is it the ideal cognitive screening test?. Int J Geriatr Psychiatry..

[39] Gaestel Y, Amieva H, Letenneur L, Dartigues JF, Fabrigoule C. (2006). Cube drawing performances in normal ageing and Alzheimer’s disease: data from the PAQUID elderly population-based cohort. Dement Geriatr Cogn Disord..

[40] Shanhu X, Linhui C, Xiaoqing J, Jing Y, Saizhu X, Ying X (2019). Effects of age and education on clock-drawing performance by elderly adults in China. Clin Neuropsychol..

[41] Costa S, St George RJ, McDonald JS, Wang X, Alty J. (2022). Diagnostic accuracy of the overlapping infinity loops, wire cube, and clock drawing tests in subjective cognitive decline, mild cognitive impairment and dementia. Geriatrics (Basel)..

[42] Pellicer-Espinosa I, Díaz-Orueta U. (2022). Cognitive screening instruments for older adults with low educational and literacy levels: a systematic review. J Appl Gerontol..

[43] Manjavong M, Limpawattana P, Sawanyawisuth K. (2021). Performance of the Rowland Universal Dementia Assessment Scale in screening mild cognitive impairment at an outpatient setting. Dement Geriatr Cogn Dis Extra..

[44] Barbosa BJAP, Siqueira Neto JI, Alves GS, Sudo FK, Suemoto CK, Tovar-Moll F (2022). Diagnóstico do comprometimento cognitivo vascular: recomendações do Departamento Científico de Neurologia Cognitiva e do Envelhecimento da Academia Brasileira de Neurologia. Dement Neuropsychol..

[45] Matías-Guiu JA, Valles-Salgado M, Rognoni T, Hamre-Gil F, Moreno-Ramos T, Matías-Guiu J. (2017). Comparative diagnostic accuracy of the ACE-III, MIS, MMSE, MoCA, and RUDAS for screening of Alzheimer disease. Dement Geriatr Cogn Disord..

[46] Schoenmakers B, Robben T. (2021). Barriers in screening for dementia in elderly migrants in primary care and the use of the Rowland Universal Dementia Assessment Scale. A mixed cross-sectional and qualitative study. Eur J Gen Pract..

[47] Voncken L, Albers CJ, Timmerman ME. (2019). Model selection in continuous test norming with GAMLSS. Assessment..

[48] Crompvoets EAV, Keuning J, Emons WHM. (2021). Bias and precision of continuous norms obtained using quantile regression. Assessment..

[49] Ribeiro FS, Teixeira-Santos AC, Leist AK. (2022). The prevalence of mild cognitive impairment in Latin America and the Caribbean: a systematic review and meta-analysis. Aging Ment Health..

[50] Chile. Instituto Nacional de Estadísticas. (2018). Síntesis de resultados. Censo 2017 [Internet]..

